# DNA-Biofunctionalization of CTAC-Capped Gold Nanocubes

**DOI:** 10.3390/nano10061119

**Published:** 2020-06-05

**Authors:** Nicole Slesiona, Sophie Thamm, H. Lisa K. S. Stolle, Viktor Weißenborn, Philipp Müller, Andrea Csáki, Wolfgang Fritzsche

**Affiliations:** Leibniz-Institute for Photonic Technology, Albert-Einstein-Straße 9, 07745 Jena, Germany; SlesionaN@cardiff.ac.uk (N.S.); sophie.thamm@leibniz-ipht.de (S.T.); lisa.stolle@leibniz-ipht.de (H.L.K.S.S.); viktor.weissenbornviktor@leibniz-ipht.de (V.W.); philipp.mueller@leibniz-ipht.de (P.M.); andrea.csaki@leibniz-ipht.de (A.C.)

**Keywords:** biofunctionalization, CTAC, shape-anisotropic nanoparticles, gold nanocubes, DNA

## Abstract

Clinical diagnostics and disease control are fields that strongly depend on technologies for rapid, sensitive, and selective detection of biological or chemical analytes. Nanoparticles have become an integral part in various biomedical detection devices and nanotherapeutics. An increasing focus is laid on gold nanoparticles as they express less cytotoxicity, high stability, and hold unique optical properties with the ability of signal transduction of biological recognition events with enhanced analytical performance. Strong electromagnetic field enhancements can be found in close proximity to the nanoparticle that can be exploited to enhance signals for e.g., metal-enhanced fluorescence or Raman spectroscopy. Even stronger field enhancements can be achieved with sharp-edged nanoparticles, which are synthesized with the help of facet blocking agents, such as cetyltrimethylammonium bromide/chloride (CTAB/CTAC). However, chemical modification of the nanoparticle surface is necessary to reduce the particle’s cytotoxicity, stabilize it against aggregation, and to bioconjugate it with biomolecules to increase its biocompatibility and/or specificity for analytical applications. Here, a reliable two-step protocol following a ligand exchange with bis (p-sulfonatophenyl) phenyl phosphine (BSPP) as the intermediate capping-agent is demonstrated, which results in the reliable biofunctionalization of CTAC-capped gold nanocubes with thiol-modified DNA. The functionalized nanocubes have been characterized regarding their electric potential, plasmonic properties, and stability against high concentrations of NaCl and MgCl_2_.

## 1. Introduction

Noble metal nanoparticles exhibit special physical and optical properties. This effect originates in the specific interaction of the metal nanoparticles upon irradiation with light. Namely, collective oscillations of the conduction band electrons are induced that are known as ‘particle plasmons’ or localized surface plasmons (LSP) [1,2]. Due to their strong interaction with light in the visible spectrum and the strong field enhancements that can be found at edges and corners of such nanostructures [3], shape-anisotropic gold nanoparticles have become an integral part in various biomedical detection devices and nanotherapeutics. The design of novel biosensors with the ability of signal transduction of biological and chemical recognition events into characteristic optical signals utilizing metal nanoparticles is of growing interest [4]. The high biocompatibility of gold nanoparticles especially facilitates their incorporation into biosensor designs that exhibit enhanced analytical performance [5]. To integrate shape-anisotropic gold nanoparticles into analytic sensing devices it is necessary to establish a reliable functionalization protocol. 

Shape-anisotropic geometry of chemically synthesized particles is usually realized by application of customized surface ligands [6,7,8,9]. These capping agents play a versatile role and are used to control size and shape, stabilize against aggregation, as well as to ensure the structural stability of the nanoparticles [10,11]. The aggregation tendency of nanoparticles based on van der Waals forces is counteracted by surface capping agents that prevent the particles from aggregation, typically based on electrostatic and/or steric stabilization [12]. However, these ligands often restrict the access of reactants to the particle surface, impeding the further functionalization of the particle, which neutralizes their otherwise useful properties. In many cases, these capping agents hamper further conjugation reactions. Removing these surfactants often results in colloidal instability and coalescence [13]. Furthermore, surface ligands render colloidal particle solutions more complex by changing the overall ionic composition of the medium. Thus, the choice of ligand influences the surface charge of the particle and the interactions between the capping molecules and possible reactants [10,14]. While gold nanoparticles themselves and the influence of their shape and size have been studied well, their outer organic shells and their effect on particle functionalization remain elusive and are the subject of extensive research. The functionalization of the particles with biomolecules is based on either surfactant replacement or conjugation of additional groups while maintaining colloidal stability [13,14,15,16,17,18,19]. Hence, mild ligand exchange reactions are needed in order to enable the biofunctionalization of gold nanoparticles. 

Oftentimes, functional biomolecules such as DNA cannot sufficiently penetrate a dense layer of capping agents or replace them. There are several protocols that have been developed for gold nanoparticle functionalization with thiol-modified DNA. Widely adapted techniques are the salt-aging method by Mirkin et al. [20] and surfactant-assisted methods that are often applied in protocols aimed at the biofunctionalization of shape-anisotropic gold nanoparticles as proposed by Li et al. [21]. Furthermore, there are viable protocols like low-pH-assisted methods as published by Zhang et al. [22], or the freezing-driven method by Liu et al. [23], and lastly a method by Lekkerker et al. who realized gold nanoparticle functionalization by depletion stabilization [24]. Surfactant-assisted methods aim at replacing surface ligands by adding an excess of small molecules with higher affinity towards the metallic nanoparticle. These small molecules make room for new and bigger ligands that contain—or are modified with—groups with even higher affinity to gold than the previous ligand [21,25]. This approach of particle functionalization was accomplished with several different nanoparticle shapes, such as nanorods, -spheres, -prisms, and -cubes (synthetized with cetyltrimethylammonium bromide (CTAB)) [20,21,22,26,27]. Aside from surfactants, a few small molecules or polymers have also been applied to facilitate DNA conjugation while avoiding gold nanoparticle aggregation. For example, Alivisatos et al. displaced citrate by using dipotassium bis(p-sulfonatophenyl) phenylphosphine dihydrate (BSPP) as an intermediate and stabilized ligand [28,29]. 

**Figure 1:**
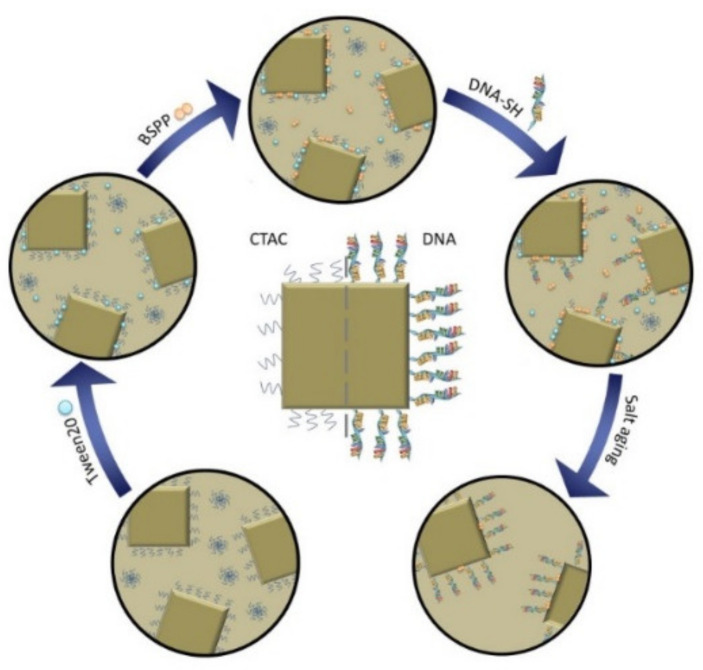
Schematic illustration of the functionalization process. Cetyltrimethylammonium chloride (CTAC) (blue squiggles) coated gold nanocubes are stabilized with Tween20 (blue spheres) to perform a ligand exchange reaction. The CTAC is thus replaced with bis (p-sulfonatophenyl) phenyl phosphine (BSPP) (orange double spheres), which both stabilizes the particles and allows the biofunctionalization with DNA as a weak ligand. A denser DNA-loading onto the particle is subsequently achieved by a salt-aging step. Here, the thiol groups gradually displace the DNA bases that are adsorbed and therefore wrapped around the gold nanoparticles and therefore hindering the access of additional DNA-strands to the particle surface. The salt-aging step neutralizes the negative DNA backbone and hydrolyzes the DNA bases that would otherwise adsorb to the nanoparticle surface.

Gold nanocubes can be easily synthesized with high precision regarding their dimensions with the help of a microfluidic synthesis set up resulting in gold nanocubes with low size distribution and an immensely reduced material consumption [30]. Their synthesis is realized with the help of cetyltrimethylammonium chloride (CTAC), which is also frequently used in batch synthesis approaches [30,31]. There is a variety of protocols suggesting different paths to exchange surface ligands like citrate and CTAB with functional, biocompatible groups [11,13,32,33,34], including the removal of CTAC ligands from the gold nanosphere’s, nanostar’s, or nanocube’s surface [35,36,37]. Current methods are either based on a solvent extraction by dichloromethane (DCM) or use an ethanol-methanol mixture or the irritant acetonitrile to remove CTAC. However, the above mentioned approaches are hardly compatible with biological applications. This problem could be resolved by CTAC removal using the salt-aging method combined with sodium dodecyl sulfate (SDS) stabilization [38]. However, this approach employs reagents that are known to express limited biocompatibility or even toxicity like CTAB and benzyldimethylhexadecylammonium chloride (BDAC). To overcome this limitation, we utilized a mild ligand-exchange to achieve a straightforward biofunctionalization of CTAC-capped gold cubes with DNA, in order to allow their utilization of these particles for bioanalytical applications. Although this applies less to purely sensing purposes, other application like the envisioned—and already partially realized (see e.g., [39])—use of particles for in vivo therapy depend on minimal cytotoxicity. The developed two-step protocol presented in this work combines the approaches of ligand-exchange with salt-aging (Figure 1). This allows for a reliable, non-invasive, and efficient conjugation of thiol-modified DNA oligonucleotides to shape-anisotropic particles with great potential for biosensing applications and nanotherapeutics.

## 2. Materials and Methods

All chemicals were purchased from Sigma-Aldrich Chemie GmbH (Taufkirchen, Germany) unless otherwise noted and were used without further purification.

### 2.1. Synthesis of Gold Nanocubes

**Figure 2:**
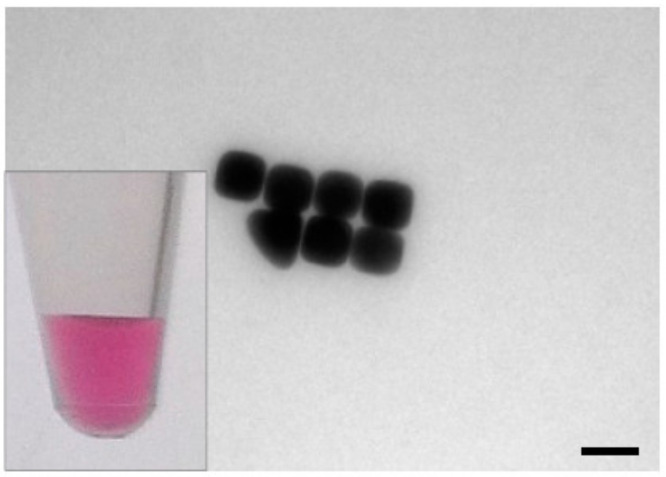
TEM image of gold nanocubes, which show a bright pink color in colloidal solution (inset: photograph of nanocube solution). The gold nanocubes had a size of 49.4 ± 5 nm with a surface area of 0.0146 µm^2^, scalebar: 50 nm.

The synthesis of gold nanocubes was carried out based on the method reported by Wu et al. [31,40]. In the first step, gold seeds were obtained by combining 0.025 M HAuCl_4_ and 0.1 M CTAC solution in a total volume of 10 mL. Subsequently, 450 µL of freshly prepared ice cold NaBH_4_ solution (0.02 M) was added under vortexing. A color change of the solution from colorless to lucent brown indicates successful seed formation. During the 1 h incubation time of the seeds at room temperature (21 °C), two batches of growth solution (GS1 and GS2), each consisting of 9.625 mL 0.1 M CTAC solution, 250 µL of 0.01 M HAuCl_4_ solution, and 10 µL 0.01 M NaBr solution, 90 µL of 0.04 M ascorbic acid solution, were prepared. After incubation, 60 µL of the seed solution was added to the first growth solution GS1 at 30 °C under shaking. After 5 s, after which a visible color change of the solution from clear to light red occurred, 60 µL of the now light red GS1 was added to the second growth solution GS2 at 30 °C under shaking for 10 s. Without further treatment, GS2 was then left for 8 min to allow reaction/particle growth during which the color of the solution turned from colorless to red/pink. Subsequently, the solution was centrifuged at 3000 rpm for 5 min. After the centrifugation step, the supernatant was removed and the nanocubes were redispersed in 10 mL ddH_2_O. Figure 2 shows a TEM image of the synthesized gold nanocubes.

### 2.2. Conjugation of Gold Nanocubes

First, the particles were washed with DEPC water 1–2 times and adjusted to an OD at the localized surface plasmon resonance (LSPR) maximum (λ_centroid_) of 5. The subsequent biofunctionalization was conducted by successive addition of 5 µL 2% aqueous Tween^®^ 20, 5 µL 0.1 M BSPP, 15 µL 0.6 mM thiol-modified capping strands for overnight incubation (5′-(ATT)_3_T_4_-(CH_2_)_3_-SH 3′ by Biomers GmbH, Ulm, Germany), 3 M NaCl in six steps of 5 × 20 µL and 1 × 25 µL to 30 µL gold nanocubes. Between the additions of each reactant, the sample was vortexed and incubated in a Thermomixer comfort (Eppendorf AG, Hamburg, Germany) at 400 rpm and RT for 20 min, followed by a final incubation for 48 h on an orbital shaker at 400 rpm and RT. Finally, the sample was purified by centrifugation at 5000× *g* for 10 min, removing the supernatant and addition of 0.02% aqueous SDS for additional stabilization. The washing step was conducted three times.

### 2.3. Zeta (ζ) Potential Measurements

ζ potential measurements were performed with a Zetasizer ZEN3600 Malvern Instruments Ltd. (Worcestershire, UK) and were conducted in disposable folded capillary cells (DTS1070). In the Zetasizer software, the parameters were to set as follows: (1) material: gold (Malvern), (2) dispersant: water at a temperature of 25 °C (viscosity 0.8872 cP, refractive index: 1.330), (3) equilibration time for temperature stabilization: 120 s, (4) measurement: 3 measurements with no delay in between with at least 10 runs per measurement. The mean of the measurements was calculated and plotted.

### 2.4. UV–VIS Spectroscopy

Gold nanocubes were spectrally characterized with a ThermoScientific^TM^ NanoDrop One (Waltham, MA, USA) and a V-670 Jasco UV–VIS/NIR spectrophotometer (Easton, MD, USA). The spectra were measured in 0.5 nm (NanoDrop) and 0.1 nm (Jasco) wavelength increments. The obtained raw data was evaluated using a custom-made Python 3.7 script. The center of gravity of the localized surface plasmon resonance (LSPR) peak (λ_centroid_) was calculated according to Dahlin et al. [41]. Peaks that were compared for validation of particle functionalization with thiol-modified DNA were normalized to improve the visual distinction between λ_centroid_ of unmodified and modified gold nanocubes. The UV–VIS range of 200–300 nm is thereby not considered in the discussion of all obtained UV–VIS spectra because of the strong signals of Tween^®^ 20 and BSPP at 231 nm and 270 nm, respectively. Due to their high intensity, they superimpose DNA peaks at 260 nm. Additionally, UV–VIS spectroscopy was used as a characterization method for colorimetric salt-induced aggregation assays.

## 3. Results and Discussion

### 3.1. Conjugation of Gold Nanocubes

To realize the binding of thiol-modified DNA oligonucleotides to gold nanocubes deriving from detergent-based synthesis, experiments testing various published functionalization methods aimed at particle biofunctionalization were conducted. The protocols were applied in their original form as published as well as with additional adjustments that are known to increase DNA-loading onto gold nanoparticles. Experiments solely based on either low-pH-assisted, salt-assisted or surfactant-assisted ligand-exchange methods did not lead to successful gold nanocube biofunctionalization (details given in Appendix A).

In ligand-exchange protocols, small surfactants are used to detach previous surfactants and make room for bulkier new ligands. When trying out different protocols, particle aggregation was observed at different stages of nanoparticle functionalization. The most promising attempts, in which particle aggregation occurred only in the late steps of the functionalization protocols, were combined to a single protocol that resulted in the successful biofunctionalization of gold nanocubes (Table 1). In conclusion, 20 min incubation time was introduced after each reactant addition step in order to prevent particle aggregation. Indeed, the particles remained stable during the whole process, and salt addition did not result in particle destabilization. The final protocol combined the salt-assisted with the surfactant-assisted method.

**Figure 3:**
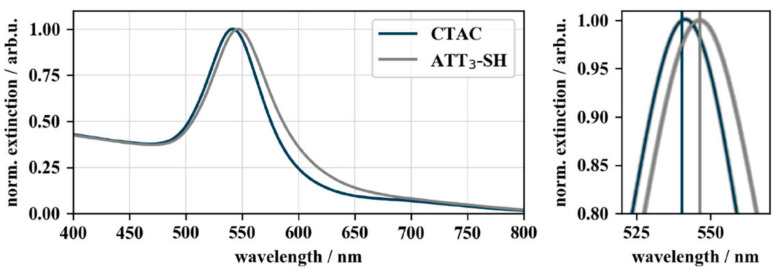
UV–VIS spectra of CTAC- and DNA-functionalized gold nanocubes. The UV–VIS spectra show the 5.3 nm wavelength shift of the localized surface plasmon resonance (LSPR) peak from 541.2 nm of CTAC-capped (blue) to 546.5 nm of DNA-capped (grey) gold nanocubes. The spectra were normalized to their peak maximum to allow a better comparison of the LSPR shift (OD_final_ = 0.26).

To confirm the successful conjugation by a shift of the LSPR peak, UV–VIS spectra of the functionalized particles were recorded. The results are displayed in Figure 3. Gold nanocubes showed a wavelength shift of 5.3 nm after bioconjugation of CTAC-capped gold nanocubes from 541.3 nm to 546.5 nm. This red-shift can be explained as a consequence of the increased local refractive index caused by the adsorption of DNA on the particle surface [42]. DNA exhibits a refractive index of 1.45 (for ssDNA at a length of 27 nt) [43] while water and CTAB (comparable to CTAC) have a refractive index of 1.33 and 1.41 [44], respectively. 

### 3.2. Characterization of the DNA-Conjugation Process Using ζ Potential Measurements

**Figure 4:**
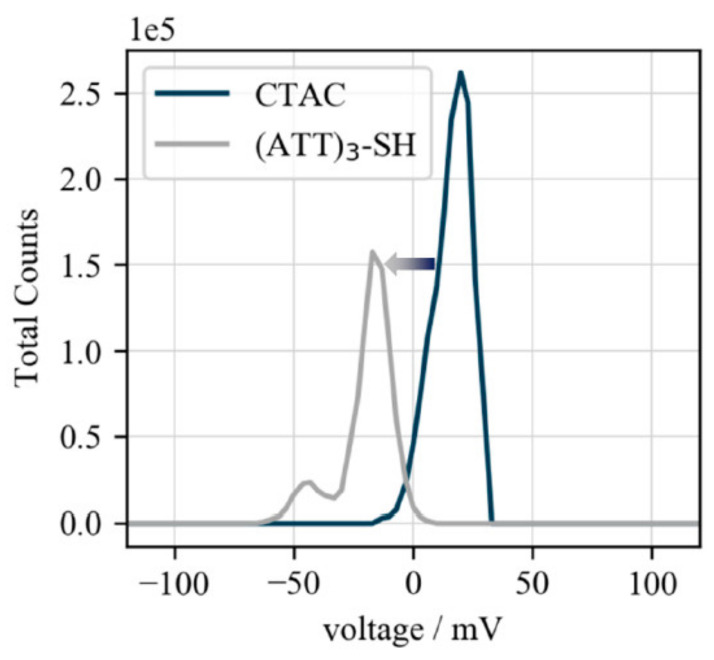
ζ potential of biofunctionalized gold nanocubes. ζ potentials of CTAC- (+20 mV) and (ATT)_3_-SH-capped (−17 mV) gold nanocubes. The shift from positive to negative confirms the replacement of the positively charged CTA+ ligands by the negatively charged DNA-strands.

The successful particle functionalization was further confirmed by ζ potential measurements. As the CTA+ ligands are positively charged, nanoparticles capped with these ligands possess a positive ζ potential, whereas following DNA-functionalization, a negative ζ potential is expected due to the negative DNA backbone. Thus, a ζ potential shift from positive (+20 mV) to negative (−17 mV) confirms the success of biofunctionalization (Figure 4).

### 3.3. Validation of DNA-Conjugation Using Colorimetric Aggregation Tests

Nanoparticles capped with their synthesis ligand cannot withstand higher salt concentrations and will aggregate [32]. The aggregation of gold nanoparticles causes the loss of their plasmonic properties and hence their characteristic color in colloidal solution. Therefore, the change in the optical density of the nanoparticle solution following salt addition can be used as a basis for a colorimetric proof of stability. This change in the extinction behavior can be measured by UV–VIS spectroscopy [45].

**Figure 5:**
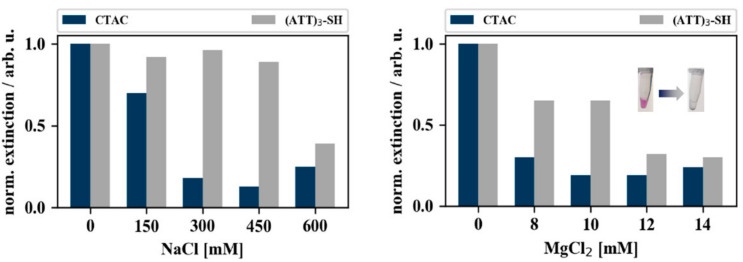
Coalescence of gold nanocubes when subjected to different concentrations of NaCl and MgCl_2_. The bar charts depict the normalized extinction intensities of CTAC-capped (blue) and (ATT)_3_-capped (grey) gold nanocubes after 30 min incubation with varying concentrations of MgCl_2_ (right) and NaCl (left). The extinction intensities were normalized to the extinction intensity of the samples that were not incubated with salt (0 mM). Typical color change upon salt addition is shown as inset.

**Figure A1:**
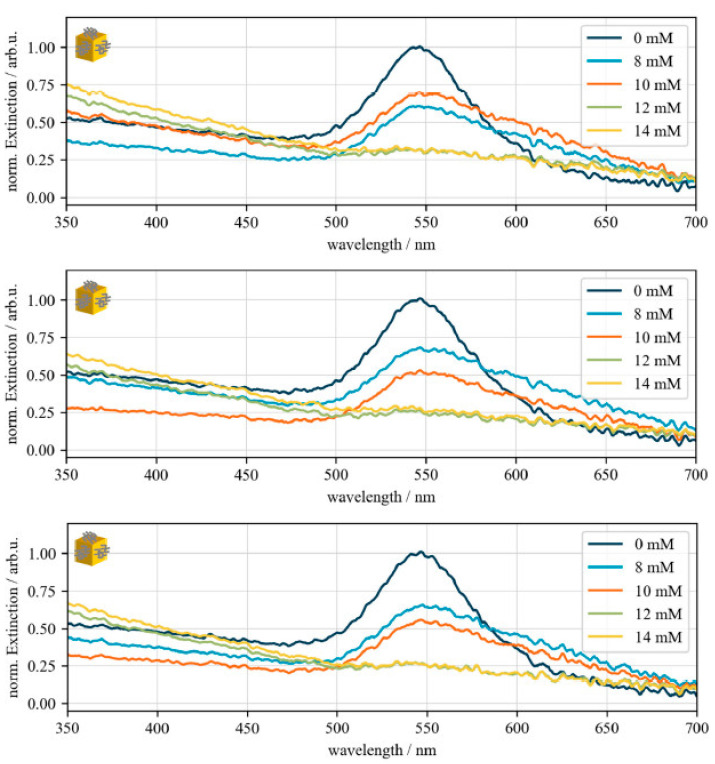
Validation of MgCl_2_-induced gold nanocube destabilization. UV–Vis spectra of different salt stability experiments of biofunctionalized gold nanocube to validate the aggregation threshold between 10 and 12 mM MgCl_2_. Dark blue: 0 Mm MgCl_2_, light blue: 8 mM MgCl_2_, orange: 10 mM MgCl_2_, green: 12 mM MgCl_2_, yellow: 14 mM MgCl2. For better comparison, the spectra were normalized to centroid of the sample that was not incubated with salt (0 mM).

Figure 5 illustrates the results of the aggregation experiments conducted with gold nanocubes, by monitoring the loss in extinction (color) of the colloidal particle solutions. CTAC-capped gold nanocubes (blue bars) aggregated right upon addition of salt. The DNA-capped gold nanoparticles (grey bars) expressed higher salt stability compared to their CTAC-capped counter parts but aggregated nevertheless when subjected to MgCl_2_ concentrations above 10 mM (additional spectra are given in Appendix B Figure A1).

## 4. Conclusions and Outlook

The paper introduces a protocol to conjugate CTAC-capped gold nanocubes with thiol-modified DNA-oligonucleotides for bioanalytical and nanotherapeutic applications. The presented method combines a mild ligand-exchange reaction with a salt-aging method under biocompatible conditions. The characterization of the functionalized particles by LSPR-shift measurement, Zeta potential measurements and colorimetric aggregation assays gives further insight into bioconjugation and stability of nanoparticles capped with CTAC.

The colorimetric aggregation assay with the utilized 13-mer DNA-oligonucleotides indicated that the conjugated particles were able to withstand up to 10 mM MgCl_2_ before particle aggregation was observed. In the future, an increased salt stability can be achieved by functionalizing the particles with longer DNA-oligonucleotide capping strands. The increased salt stability of gold nanocubes was realized by the additional electrostatic stabilization due to the additional charging of the DNA backbone and the additional steric shielding from the external environment. The presented results show a reproducible alternative conjugation technique for shape-anisotropic plasmonic nanoparticles. The two-step ligand-exchange reaction under mild biocompatible conditions allows the use of such conjugates for bioanalytical applications with high biocompatibility with a yield of 25%. Although the ionic stability of the particles is not yet sufficient for direct cellular applications, knowledge of this concentration threshold is paramount when using these conjugates as nanocomponents for biomedical detection devices and nanotherapeutics. Here, the buffer composition is of great importance and must meet various requirements, e.g., for the assembly of DNA superstructures. Especially DNA origami-based nanosensors hold great potential for smart biosensing applications in the future [46,47].

## Figures and Tables

**Table 1:** Protocols and adjustments in gold nanocube functionalization. The table lists the approaches to conjugate gold nanocubes, marking the point at which irreversible particle aggregation occurred. √ marks no aggregation at the given point, × marks irreversible aggregation. The salt concentrations express the end concentration of NaCl in the sample after all steps of addition (ligand exchange by Liu et al. in 2015).

Protocol	Comments, Additional Adjustments		Stability of Gold Nanocubes			LSPR (λ_centroid_) Shift
			In Reaction Mixture	During Incubation	After Purification	
Ligand exchange	Salt concentration and temperature					
	0.5 M NaCl	25 °C	√	×	-	-
		55 °C	√	×	-	-
	0.25 M NaCl	25 °C	√	×	-	-
		55 °C	√	×	-	-
	0 M NaCl	25 °C	√	√	√	×
Final protocol			√	√	√	√

## Appendix A

Several attempts to functionalize gold nanocubes were made. Different functionalization protocols that were originally designed for functionalization of gold nanorods and -spheres were tested for their applicability to gold nanocubes. The protocols were tried out several times with slight adjustments of either pH, salt concentration, or incubation conditions.

The first approach that was tested was the pH-assisted method by Zhang et al. that is usually applied in gold nanosphere biofunctionalization. Minor adjustments were made, as functionalization protocols of anisotropic nanoparticles generally suggest higher concentrations of capping strands to achieve biofunctionalization. Hence, 13 µL of 0.6 M capping strands, 200 µL of CTAC-capped Gold nanocubes, and 87 µL DEPC-treated H2O were added together in a 0.5 mL reaction tube and incubated for 30 min on a Thermomixer at RT and 300 rpm. Following incubation, 6 µL of 0.5 M citrate buffer (pH 3, adjusted with 37% HCl) were added. After another incubation cycle of 30 min on a Thermomixer at RT and 300 rpm, 41.7 µL 5 M NaCl were added (end concentration of 0.6 M NaCl) and the solution was incubated for 60 min in a Thermomixer at RT and 300 rpm. In a second try, half of the above-mentioned salt concentration was used.

Salts like NaCl are used in gold nanoparticle DNA-functionalization protocols to overcome the electrostatic repulsion of the DNA backbone and to increase the density of the final DNA-layer around the particle. Yet, gold nanocubes capped with CTAC express a positive charge. When NaCl is added to the gold nanocubes, the Debye length between the particles shortens due to charge compensation of the added Cl- ions. If the interparticle repulsion decreases to a certain point, strong van der Waals forces between the gold nanoparticles cause their aggregation, rendering the particles unusable for further experiments. This ambivalent characteristic makes salt addition both necessary but also undesirable. This problem has been solved by Mirkin et al. who proposed a so-called salt-aging method. Here, salts are added gradually over a time period of 24 h to provide the means of slowly functionalizing the gold nanoparticles with DNA by decreasing the electrostatic repulsion between the capping strands, while not causing colloidal instability by rapidly increasing the salt concentration.

Following the salt-assisted method, 7.5 µL gold nanocubes were mixed with 7.5 µL of 0.6 M thiol-modified capping strands and 85 µL of DEPC-treated H2O. After 30 min incubation at RT, 4 µL of 0.5 M citrate buffer were added followed by another incubation step of 45 min at RT. Subsequently, 2 µL 1 M NaCl were added to the sample. After 3 h of incubation at RT, 2.5 µL 5 M NaCl were pipetted into the reaction mixture. After a 3 h incubation step, 3.8 µL 5 M NaCl were added. Finally, the sample was centrifuged at 4000× *g* for 10 min to remove the supernatant and resuspended the particles in DEPC-treated H2O. Additional adjustments that were made to this protocol are stated in.

A method that was proposed by Liu et al. in 2015 approaches biofunctionalization of shape-anisotropic gold nanoparticles by first exchanging the gold nanoparticles capping of CTAB partially with a smaller molecule to achieve better accessibility to the particle surface (surfactant-assisted method). For example, surfactants like Tween^®^ 20 and SDS initially adsorb on the particle surface, stabilizing them against low salt concentrations. The proposed protocol was slightly adjusted for Gold nanocubes functionalization. Then, 5 µL of the nonionic surfactant Tween^®^ 20 (2%, aqueous solution) was used to stabilize 30 µL gold nanocubes solution. Subsequently, 5 µL 0.1 M BSPP were added. BSPP is a small molecule that can penetrate the dense CTAB layer of gold nanorods and additionally stabilize the particles with its two negative charges. The same behavior was expected towards the CTAC layer of gold nanocubes. A total of 15 µL of 0.6 M thiol-modified DNA were added to the sample followed by addition of NaCl to an end concentration of 0.5 M. Following a 24 h incubation at RT and 900 rpm on a Thermomixer, the sample was centrifuged for 10 min and 6000 g, and the supernatant replaced by 0.02% aqueous SDS solution. The washing step was repeated twice. Any additional adjustments that were made to this protocol are listed in the table included in the manuscript.
